# Scoring Systems in Pyogenic Spondylodiscitis—A Narrative Review

**DOI:** 10.3390/diagnostics16213511

**Published:** 2026-10-27

**Authors:** Ioana Diandra Miftode, Ionela Larisa Miftode, Andreea Emanuela Lungu, Corina Lupașcu Ursulescu, Egidia Gabriela Miftode, Cristina Furnica

**Affiliations:** 1 Department of Radiology and Medical Imaging, “St. Spiridon” County Emergency Clinical Hospital, 700115 Iași, Romania; miftode_ioana-diandra@d.umfiasi.ro (I.D.M.);; 2 “Grigore T. Popa” University of Medicine and Pharmacy, 700115 Iași, Romania; 3 “St. Parascheva” Clinical Hospital of Infectious Diseases, 700115 Iași, Romania; 4 Department of Neurology, “Prof. Dr. Nicolae Oblu” Emergency Clinical Hospital, 700115 Iași, Romania; 5 Institute of Forensic Medicine, 700115 Iași, Romania

**Keywords:** clinical decision-making, external validation, prognostic models, spinal infection, surgical intervention, vertebral osteomyelitis

## Abstract

**Background**: Pyogenic spondylodiscitis has become increasingly common over the past two decades, and its nonspecific presentation, together with frequent diagnostic delay, makes severity assessment, prognosis, and surgical decision-making difficult to standardize based on clinical judgement alone. **Methods**: This narrative review was based on a targeted literature search through August 2026, combining terms for spondylodiscitis and vertebral osteomyelitis with the names of individually identified scoring systems and validation-related terms, distinguishing development from reliability testing and independent external validation. **Results**: Several disease-specific tools addressing severity, prognosis, or the decision to operate were identified. The most extensively evaluated treatment-decision tool showed moderate-to-good, if variable, performance across cohorts, while another frequently cited score performed less consistently in an independent population. One severity classification showed confirmed reliability but more limited predictive evidence, and mortality-prediction tools remain independently unvalidated. **Conclusions**: No single system currently demonstrates consistent performance across severity assessment, prognosis, and surgical decision-making. Existing tools should therefore be applied within the specific clinical context for which they were developed, while further independent external validation is needed before broader clinical implementation.

## 1. Introduction

Spondylodiscitis is an infection of the intervertebral disc and the adjacent vertebral endplates that, once established, can extend into the paravertebral soft tissues and epidural space. Long regarded as an uncommon condition, it has become substantially more frequent over the past two decades, a trend now documented consistently across several national surveillance datasets. Population-based data from Germany show the annual incidence of pyogenic spondylodiscitis roughly doubling between 2005 and 2021, from 5.4 to 11.0 cases per 100,000 population, with in-hospital mortality rising over the same period to an overall rate of approximately 5% and disproportionately affecting patients over 80 years of age [1]. A comparable trajectory has been reported elsewhere in Europe: in France, a nationwide cross-sectional study of hospitalized adults aged 15 years and older found the incidence of vertebral osteomyelitis rising from 6.1 to 11.3 cases per 100,000 population between 2010 and 2019, with a mean incidence of 7.8 per 100,000 over the study period [2]; in England, national hospital episode statistics showed admissions for spondylodiscitis increasing from 3 to 4.4 cases per 100,000 population between 2012/13 and 2020/21 [3]. This convergent rise across independent health systems has been attributed to an ageing population, increasing use of spinal instrumentation and long-term vascular access, and a growing pool of immunosuppressed and comorbid patients. Men are affected roughly one-and-a-half to two times more often than women, and the lumbar spine is the most frequently involved segment, accounting for just over half of cases, followed by the thoracic and, less commonly, the cervical spine [1].

Bacterial (pyogenic) infection accounts for the vast majority of cases. *Staphylococcus aureus*, including methicillin-resistant strains, stands out as the most frequently identified pathogen overall, irrespective of whether spinal hardware is involved. Coagulase-negative staphylococci and *Cutibacterium acnes*, by contrast, are more typically associated with instrumentation-related infections, while *Staphylococcus epidermidis* appears disproportionately in patients undergoing long-term hemodialysis [4,5].

The spine can become infected through one of three pathways: hematogenous spread via the segmental arteries feeding the vertebral endplates—by far the most frequent mechanism; direct contamination during spinal surgery, injections, or trauma; or extension from a nearby infected site, such as a psoas abscess [4]. It is important to distinguish pyogenic spondylodiscitis from its granulomatous and fungal counterparts—tuberculous (Pott’s disease), brucellar, and fungal forms—which follow different clinical courses, tend to affect different patient populations, and call for different treatment durations. In areas where these forms remain endemic, they can represent a considerable proportion of cases that get misclassified and treated as pyogenic disease when mycobacterial cultures and serological testing are not performed [4].

Across different patient cohorts, the recognized risk factors all point to the same underlying theme: conditions that either raise the number of bacteria entering the bloodstream or lower the host’s ability to fight off infection. Recurrently cited predisposing factors include diabetes mellitus, long-term steroid use or other forms of immunosuppressive therapy, intravenous drug use, chronic kidney disease and hemodialysis, and a remote source of infection—such as endocarditis, a urinary tract infection, or an indwelling vascular catheter [4,6,7].

From a clinical standpoint, there is no single presentation that defines spondylodiscitis. Back pain is the symptom patients report most reliably, yet fever does not always accompany it, neurological deficits show up in only a subset of patients, and the textbook combination of back pain, fever, and neurological signs is actually uncommon rather than the norm [4,6]. Because the symptoms are so nonspecific—and because spondylodiscitis is far rarer than the many other, more mundane causes of back pain—there is typically a long stretch of time between when symptoms first appear and when the diagnosis is finally made. That delay carries real consequences: it is linked to worse outcomes overall, including higher rates of neurological impairment and treatment failure [6].

Taken together, these features make spondylodiscitis challenging to manage on the basis of clinical judgement alone: incidence is rising, several distinct etiologies can present similarly, clinical manifestations are often nonspecific, and diagnosis is frequently delayed. In this context, validated and clinically relevant scoring systems may provide particular value by helping clinicians assess disease severity, estimate prognosis, and identify patients who may require surgical intervention, thereby supporting greater consistency in clinical decision-making [4,6]. Several scoring systems for spondylodiscitis have been proposed over the past decade.

Despite the increasing number of proposed scoring and classification systems, no single tool integrating clinical, laboratory, and imaging parameters has undergone robust and broad external validation in pyogenic spondylodiscitis. Several systems—including BSDS, SITE, SSC/SponDT, Pola, and MSI-20—have been proposed for different purposes, particularly severity assessment and surgical decision-making; however, their reported performance and degree of external validation vary considerably. Meanwhile, well-conducted prognostic cohorts have consistently identified similar risk factors, including longer symptom duration, sepsis, neurological involvement, epidural or paravertebral abscesses, thoracic or multilevel disease, certain causative organisms, impaired renal function, marked inflammation, and comorbidities such as diabetes or immunosuppression [7,8].

Scoring systems can help guide and unify decision-making in complex clinical scenarios, providing a direction for treatment when decisions must be made swiftly and without the benefit of a multidisciplinary expert panel. Existing scoring systems tend to emphasize particular subgroups of patients—for instance those with spondylodiscitis specifically, or those with epidural abscesses—and so may not be sufficiently comprehensive for wider clinical acceptance; several have in fact been found unreliable when tested in externally validated patient cohorts. This may be because some of these systems were created for very specific clinical scenarios and lose relevance when extrapolated to the broader setting of spinal infectious disease.

Accordingly, this review aims to identify and critically appraise the scoring systems and classification tools proposed for pyogenic spondylodiscitis, with particular emphasis on their intended clinical purpose, development methodology, and level of validation. Specifically, we examine their applicability to disease severity assessment, outcome prediction, and surgical decision-making, and evaluate whether their reported performance has been confirmed in independent patient populations.

## 2. Methods

This narrative review was based on a targeted PubMed search conducted up to August 2026. The search combined terms related to “spondylodiscitis,” “vertebral osteomyelitis,” and “spinal infection”, with terms related to scoring and classification systems, including “score,” “scoring system,” “classification,” “prediction model,” and “prognostic model.” Additional searches were performed using the names and acronyms of individually identified scoring systems, including SponDT, the Pola classification, the Spondylodiscitis Severity Code (SSC), the Brighton Spondylodiscitis Score (BSDS), the Spinal Infection Treatment Evaluation (SITE) Score, the Spinal Instability Spondylodiscitis Score (SISS), the MRI Spondylodiscitis Severity Index (MRI-SSI), and the SPONDY-Score [9], combined with validation-related terms such as “external validation,” “reliability,” “calibration,” “ROC,” “AUC,” “sensitivity,” and “specificity.”

The review focused primarily on scoring and classification systems developed for or evaluated in adults with pyogenic spondylodiscitis, native vertebral osteomyelitis, or de novo spinal infection. Studies enrolling broader or mixed spinal infection populations were also considered when they provided relevant data on the development, application, or validation of an identified scoring system; the composition of these cohorts was taken into account when interpreting the evidence. Tuberculous, brucellar, and fungal spondylodiscitis were not considered equivalent to pyogenic disease and were excluded from the primary assessment whenever results could be distinguished by etiology. Tools originally developed for other conditions, such as the Spinal Instability Neoplastic Score (SINS), were included only when they had been formally evaluated in spinal infection cohorts or used as comparators against disease-specific scoring systems; such evidence was considered separately and was not regarded as validation of a spondylodiscitis-specific tool. A related framework, Stability, Neurology, Infection, and Morbidity (SNIM), was also identified [10]; because the full text was not accessible for the present review, it was not included in the detailed comparative assessment.

For each identified scoring or classification system, we assessed its intended clinical purpose, derivation population, target outcome, and available evidence of validation. Score development, internal validation, external predictive validation, and inter- or intra-observer reliability were considered distinct forms of evidence. External validation was defined as assessment of score performance in a patient population independent of the original derivation cohort, whereas reliability analyses were considered evidence of reproducibility rather than predictive validity. Where available, measures of discrimination, calibration, sensitivity, specificity, and inter- or intra-observer reliability were considered when evaluating the performance and level of validation of each tool. Generative artificial intelligence (GenAI) tools were used exclusively to assist with English language editing and to improve the clarity and readability of the manuscript; please refer to Supplementary Material File S1 for a detailed AI disclosure statement.

## 3. Results

### 3.1. Treatment-Decision Scores

Antimicrobial therapy remains the cornerstone of treatment for pyogenic spondylodiscitis, while surgical intervention is required in selected patients. Beyond the presence of significant neurological deficits, however, indications for surgery are not uniformly defined, and substantial variation in treatment decisions has been reported among European spine surgeons [11]. A systematic review and meta-analysis reported a survival benefit associated with early surgical treatment compared with conservative management [12], and a propensity score-matched cohort analysis similarly found lower mortality among surgically treated patients [13]. In contrast, the multicenter 2SICK study of septic patients with primary spondylodiscitis reported lower mortality among patients undergoing delayed surgery than among those treated with early surgery or conservative therapy [11]. These differing findings highlight the uncertainty surrounding surgical decision-making in pyogenic spondylodiscitis and provide a rationale for the development of structured tools intended to support treatment selection.

#### 3.1.1. Brighton Spondylodiscitis Score (BSDS)

The Brighton Spondylodiscitis Score (BSDS) was developed by Appalanaidu et al. to identify patients with spondylodiscitis at increased risk of requiring surgical intervention [14]. The score was derived retrospectively from a cohort of 65 patients, of whom 21 underwent surgery, following evaluation of 35 candidate variables. Six domains were incorporated into the final model: distant site infection, medical comorbidity, immunocompromised status, MRI findings, anatomical location, and neurological impairment. The resulting score ranges from 6 to 35 points and stratifies patients into low-, moderate-, and high-risk categories, with increasing scores corresponding to a higher predicted likelihood of surgical intervention [14].

The original model showed good discrimination in the derivation cohort (AUC 0.83) and was subsequently tested in an additional 20-patient cohort, in which discrimination decreased to an AUC of 0.71 [14]. Subsequent independent evaluations have reported variable performance, raising concerns regarding its generalizability.

#### 3.1.2. SITE Score

The Spinal Infection Treatment Evaluation (SITE) Score was introduced by Pluemer et al. as a structured decision-support tool for the management of de novo spinal infections [15]. Developed through literature review and iterative assessment by an international multidisciplinary expert panel, the score incorporates five domains: neurological status, location of infection, radiological findings related to instability and neural-element involvement, pain severity, and host comorbidities. Total scores range from 0 to 15 points, with lower scores reflecting greater disease severity and a stronger recommendation for surgical management [15]. The initial proof-of-concept study demonstrated high inter- and intra-observer reliability, although testing was performed in a small set of selected cases and primarily assessed agreement with expert treatment decisions rather than subsequent clinical outcomes [15].

Subsequent studies evaluated the SITE Score in independent clinical cohorts. Xiong et al. assessed its performance in 213 patients with de novo spinal infections and reported an AUC of 0.743 (95% CI 0.67–0.81) for discriminating between operative and non-operative management [16]. In a comparative cohort of 148 patients, Rezvani et al. reported an AUC of 0.86 for SITE, compared with 0.81 for the Pola classification and 0.73 for BSDS; a SITE cutoff of 8.5 yielded a sensitivity of 80.6% and specificity of 81.2% for operative treatment [17]. More recently, Britt et al. externally evaluated the score in 95 patients with de novo spinal infections and found 82.1% concordance between SITE-based recommendations and actual treatment decisions, with moderate discrimination for operative versus conservative management (AUC 0.70) [18].

Overall, SITE has undergone evaluation across several independent cohorts, with generally favorable but variable performance. Importantly, these studies primarily assessed concordance with or discrimination of actual treatment decisions rather than an independently adjudicated need for surgery. The implications of this distinction, together with the variability in performance across external cohorts, are discussed further in Section 3.5.

#### 3.1.3. SINS as a Comparator in Spinal Infection

The Spinal Instability Neoplastic Score (SINS) was originally developed to assess mechanical instability in patients with neoplastic spinal disease and is therefore not a disease-specific scoring system for spondylodiscitis. Nevertheless, it has been evaluated as a comparator in patients with de novo spinal infection. In a comparative study of 123 patients, Pluemer et al. assessed SINS alongside the SITE Score and SISS against multidisciplinary expert treatment recommendations [19]. SINS showed lower agreement with the expert panel than the infection-specific tools, supporting the limited applicability of a scoring system developed for neoplastic instability when extrapolated to spinal infection [19].

### 3.2. Severity-Assessment and Classification Systems

#### 3.2.1. The Pola Classification

The Pola classification is a clinical–radiological system developed to stratify pyogenic spondylodiscitis according to disease severity and to provide a corresponding treatment algorithm [20]. It was developed from a cohort of 250 patients treated between 2008 and 2015 and incorporates three principal features: bone destruction or segmental instability, epidural abscess formation, and neurological impairment [20]. Patients are classified into three major categories: type A, characterized by the absence of biomechanical instability, epidural abscess, and neurological impairment; type B, characterized by significant bone destruction or biomechanical instability without epidural abscess or neurological impairment; and type C, defined by the presence of epidural abscess and/or neurological compromise. Further subtypes refine the classification and are linked to progressively more intensive treatment strategies [20].

Although the original study described the system as a validation study and reported clinical outcomes over a two-year follow-up, development and evaluation were performed within the same 250-patient cohort [20]. Accordingly, this evidence is more appropriately considered part of the initial development and clinical evaluation of the classification rather than independent external validation. Subsequent independent work has demonstrated reproducibility of the Pola classification, including assessment of inter-observer reliability [21]. Its ability to discriminate operative from non-operative management has also been evaluated in a separate 148-patient cohort [17], although this represents a different construct from reliability.

#### 3.2.2. Spinal Instability Spondylodiscitis Score (SISS) and Modified SISS (mSISS)

The Spinal Instability Spondylodiscitis Score (SISS) was developed specifically to assess mechanical instability in spontaneous spondylodiscitis and to support treatment decisions related to spinal stabilization [22]. Derived by expert consensus and initially evaluated in a retrospective cohort of 127 patients, the score incorporates four domains—extent of bone involvement, spinal alignment, anatomical location, and mechanical pain—and classifies affected segments as stable, potentially unstable, or unstable. The original study demonstrated excellent inter-observer reliability for the total score (ICC 0.960) and reported better performance than SINS in identifying instability in the setting of spinal infection [22].

Subsequent evaluation, however, highlighted the distinction between assessment of mechanical instability and prediction of operative treatment. In an independent cohort of 213 patients with de novo spinal infection, SISS showed substantial inter-rater reliability (ICC 0.68, 95% CI 0.56–0.76), but poor discrimination between patients managed operatively and non-operatively (AUC 0.557, 95% CI 0.47–0.64); mean SISS scores did not differ significantly between the two treatment groups [16]. These findings suggest that reproducible assessment of infection-related instability does not necessarily translate into accurate prediction of overall surgical management.

The SISS was subsequently revised through expert consensus within the AO Spine Trauma and Infection Knowledge Forum, resulting in the modified SISS (mSISS) [23]. The revised score retains four domains—location, extent of bone lesion, spinal alignment, and mechanical pain—but simplifies their definitions, including assessment of bone involvement at the affected spinal segment rather than at the individual vertebral-body level. In a multicenter reliability study, 15 experienced spine surgeons independently assessed ten representative cases on two occasions. The mSISS demonstrated excellent intra-rater reliability for the total score (ICC 0.90, 95% CI 0.84–0.96) and high inter-rater reliability in both assessment rounds (ICC 0.87 and 0.89, respectively) [23]. These findings support the reproducibility of mSISS for classifying mechanical instability; however, its predictive performance against clinical outcomes or operative management has not yet been independently established.

#### 3.2.3. MRI Spondylodiscitis Severity Index (MRI-SSI)

The MRI Spondylodiscitis Severity Index (MRI-SSI) was developed as an imaging-based tool for assessing disease burden and severity in patients with pyogenic spondylodiscitis [24]. The score is derived from contrast-enhanced MRI findings and incorporates vertebral destruction, signal abnormalities, endplate erosion, and epidural or paravertebral extension. According to the total score, patients are classified into mild (0–9 points), moderate (10–19 points), or severe disease (≥20 points) [24].

In the original derivation cohort, MRI-SSI showed moderate discrimination for subsequent surgical treatment, with an AUC of 0.72; a cutoff of ≥20 yielded a sensitivity of 58% and specificity of 88% [24]. Higher MRI-SSI values were associated with greater disease severity and a higher frequency of operative management. However, these predictive estimates were obtained from the same cohort in which the score was developed, and independent external validation has not yet been reported. Accordingly, MRI-SSI should currently be regarded as a promising imaging-based severity tool with preliminary predictive evidence rather than an externally validated treatment-decision model.

#### 3.2.4. The Spondylodiscitis Severity Code (SSC)

The Spondylodiscitis Severity Code (SSC) was developed by Homagk et al. as a multidimensional classification system intended to stratify the severity of non-specific spondylodiscitis and support treatment selection [25]. Unlike purely imaging-based classifications, the SSC integrates clinical, laboratory, and radiological parameters to characterize disease severity and assign patients to corresponding therapeutic pathways. The proposed framework distinguishes increasing levels of surgical severity, with recommendations ranging from dorsal stabilization alone to combined dorso-ventral stabilization with decompression, according to the overall clinical and structural involvement [25]. Patients managed conservatively were not included in the original classification analysis.

### 3.3. Diagnostic and Treatment-Monitoring Scores

#### 3.3.1. SPONDY-Score

The SPONDY-Score is an MRI-based diagnostic scoring system developed by Albano et al. to differentiate spondylodiscitis from degenerative spinal disease in patients with vertebral endplate abnormalities [9]. In a retrospective cohort including 126 patients with spondylodiscitis and 162 with degenerative spinal disease, MRI examinations were independently assessed by two readers for 12 imaging features. After excluding collections, which were considered highly suggestive of infection, five MRI features were selected through logistic regression and incorporated into the score: posterior-element bone edema, vertebral body destruction, paravertebral edema, T2 hypointensity of the intervertebral disc, and non-anatomical T2 hyperintensity of the disc [9]. 

The resulting score assigns weighted values to these features, with a threshold of ≥5 points indicating spondylodiscitis. The dataset was randomly divided into training and testing sets (75:25). When applied to the testing set and combined with the presence of paravertebral collections as an indicator of infection, the model achieved an accuracy of 89%, sensitivity of 95%, and specificity of 85%. Inter-observer reproducibility of the individual MRI features was also favorable, with Cohen κ values ranging from 0.61 to 0.90 and raw agreement from 85% to 98% [9].

Although these findings indicate promising diagnostic performance, the testing cohort was derived from a random split of the original dataset rather than from an independent external population. Accordingly, the SPONDY-Score currently has evidence of internal testing and imaging-feature reproducibility, but independent external validation has not yet been established. Its role is primarily diagnostic and should therefore be distinguished from MRI-based tools such as MRI-SSI, which were developed to assess disease severity and predict subsequent clinical management.

#### 3.3.2. SponDT

The SponDT (Spondylodiscitis Diagnosis and Treatment) score was developed by Homagk et al. from a cohort of 296 patients treated between 1998 and 2013 as a disease-specific tool for the assessment and longitudinal monitoring of spondylodiscitis [26]. The score integrates three parameters representing complementary dimensions of disease activity: serum C-reactive protein (CRP), pain intensity assessed using a numerical rating scale (NRS), and MRI findings. These variables are combined into a composite score intended to reflect disease severity and its evolution during treatment [26].

Rather than functioning as a single-time-point predictor of operative management, SponDT was designed primarily to support diagnosis, assess disease activity, and monitor treatment response. In the original cohort, patients with severe disease had a mean SponDT score of approximately 5.6 at diagnosis, while a score below 3 was proposed as a therapeutic target before discharge [26]. The longitudinal nature of the score therefore distinguishes it from treatment-decision tools such as BSDS and SITE, which were developed primarily to stratify patients according to operative management.

However, the available evidence for SponDT derives predominantly from its original development and clinical application. Independent external validation against predefined clinical outcomes, including treatment failure, neurological deterioration, operative intervention, or mortality, has not been established in the literature identified in this review. Accordingly, SponDT is best regarded as a disease-specific assessment and monitoring tool rather than an externally validated prognostic or treatment-decision model.

### 3.4. Prognostic Models for Long-Term Mortality

Zadran et al. evaluated long-term mortality in a retrospective cohort of 125 patients with vertebral osteomyelitis managed either surgically or conservatively, with a minimum follow-up of two years [27]. Using LASSO-penalized Cox regression, the authors identified the Charlson Comorbidity Index (CCI), cardiovascular disease, CRP normalization, thoracic infection, and Karnofsky performance status as relevant predictors of mortality. The resulting prognostic model showed moderate discrimination, with reported AUC values ranging from 0.74 to 0.77 [27].

Mortality did not differ significantly between surgically and conservatively managed patients, despite greater comorbidity and disease severity among those undergoing surgery. CRP normalization within 14 days was associated with lower mortality, whereas baseline CRP could not be consistently incorporated because of incomplete measurements [27]. Although the study provides relevant information regarding prognostic factors in vertebral osteomyelitis, the model was derived from a relatively small retrospective cohort and has not undergone independent external validation. It should therefore be distinguished from a clinically implemented disease-specific scoring system.

#### Mortality in Spinal Infection (MSI-20) Score

The Mortality in Spinal Infection (MSI-20) score was developed by Lener et al. as a preoperative prognostic tool for mortality in patients with spinal infection [28]. The score was derived retrospectively from 252 patients and incorporates eight parameters: body mass index, ASA classification, presence of sepsis, age-adjusted Charlson Comorbidity Index, renal failure, hepatopathy, neurological deficit, and CRP level at diagnosis. Weighted values assigned to these variables produce a maximum score of 20 points, with higher scores indicating greater predicted mortality risk [28].

In the original cohort, an MSI-20 threshold of ≥11 identified a subgroup with particularly high mortality risk, leading the authors to propose the score as an adjunct to preoperative risk stratification and surgical decision-making [28]. However, MSI-20 was developed in a broader spinal infection population rather than exclusively in patients with pyogenic spondylodiscitis, and its reported performance derives from the original retrospective cohort. Independent external validation has not been established in the literature identified in this review. Consequently, MSI-20 should currently be regarded as a promising mortality risk-stratification tool rather than a validated instrument for determining surgical eligibility.

Beyond formal scoring systems, observational cohorts have identified several reproducible predictors of adverse outcome. In a retrospective UK cohort of 91 patients with pyogenic spondylodiscitis, Widdrington et al. reported 7% mortality and found diabetes mellitus, neurological impairment at presentation, longer symptom duration before diagnosis, and radiological evidence of spinal cord or cauda equina compression to be independently associated with an unfavorable outcome [29]. These findings illustrate the broader prognostic literature in pyogenic spondylodiscitis, in which individual risk factors are considerably better characterized than externally validated multivariable prediction scores.

### 3.5. External and Independent Validation: The Central Finding

As this review aims to examine not only the development but also the validation of the available scoring systems, particular emphasis should be placed on their performance in populations other than those used for their original derivation. While the results reported by the developers provide important information regarding the initial construction and performance of each score, independent external validation offers a more robust assessment of its reproducibility and potential clinical applicability. The available evidence is therefore summarized below in Table 1 for those scoring systems that have undergone sufficient independent evaluation to permit meaningful comparison.

The principal finding of this review is that the evidence supporting currently available scoring systems is highly heterogeneous, not only in magnitude but also in the type of validation performed. Development-cohort performance, inter-observer reliability, internal testing, and independent external predictive validation represent distinct forms of evidence and should not be interpreted interchangeably. Among the tools identified, only a limited number have been evaluated in patient populations independent of their original development cohorts, and external testing has frequently resulted in lower predictive performance than initially reported.

The BSDS provides the clearest example of this loss of performance across populations. The original study reported good discrimination in the derivation cohort (AUC 0.83), with lower performance in an additional 20-patient cohort (AUC 0.71) [14]. Subsequent independent evaluations produced heterogeneous results. Rezvani et al. reported an AUC of 0.73 in their 148-patient comparative cohort [17], whereas Urrutia et al. found essentially no discriminatory ability in an independent cohort, with an AUC of 0.469 [30]. Thus, although BSDS was developed specifically to identify patients likely to undergo surgical intervention, its predictive performance has not remained stable across independent populations.

The SITE Score currently has the most consistent external evidence among the treatment-decision tools reviewed, although its performance has varied across independent cohorts. Following favorable results in the initial proof-of-concept study [15], subsequent independent evaluations reported AUC values of 0.743 in the cohort of Xiong et al. [16], 0.86 in the comparative study by Rezvani et al. [17], and 0.70 in the external validation by Britt et al. [18]. Collectively, these studies support moderate-to-good discrimination across different populations, but they do not establish uniformly strong predictive performance. Furthermore, the principal endpoint in these studies was operative versus non-operative management or concordance with expert treatment recommendations, rather than an independently adjudicated determination of surgical necessity. Consequently, the SITE Score is best considered the treatment-decision tool with the most extensive external evaluation to date, rather than a definitively validated standard for determining the need for surgery.

For the remaining systems, the evidence is more limited or addresses different forms of validity. The Pola classification has demonstrated independent inter-observer reliability [21] and has been evaluated against operative management in a separate cohort [17], but broad external predictive validation remains limited. SISS has demonstrated reproducibility for assessing mechanical instability but showed poor discrimination for operative management in an independent cohort (AUC 0.557) [16,22], illustrating that reliability for one clinical construct does not necessarily imply predictive validity for another. The modified SISS has shown high inter- and intra-observer reliability [23], but outcome-based external validation has not yet been established. Similarly, MRI-SSI demonstrated moderate discrimination for subsequent surgical treatment in its development cohort (AUC 0.72) [24], but independent external validation has not yet been reported. SponDT and SSC remain supported predominantly by development and descriptive clinical data [25,26], while the SPONDY-Score has undergone internal testing but not independent external validation [9].

Taken together, the available evidence indicates that no currently available scoring system has yet demonstrated robust, reproducible performance across multiple independent populations and across the full range of clinically relevant outcomes. Importantly, the absence of external predictive validation should not be interpreted as evidence that a tool lacks clinical value; rather, it indicates that its generalizability remains uncertain. The distinction is particularly relevant for systems such as Pola and mSISS, for which reproducibility has been demonstrated, but whose ability to predict patient outcomes or treatment requirements remains incompletely established.

The primary purposes and main variables incorporated into the reviewed scoring systems and classifications are summarized in Table 2. Their main advantages and limitations are compared in Table 3.

## 4. Discussion

The available literature demonstrates that the term “validated” is applied to substantially different forms of evidence across scoring systems for pyogenic spondylodiscitis. A tool may demonstrate good inter-observer reliability without having established predictive validity, while favorable discrimination in a derivation cohort does not ensure comparable performance in an independent population. This distinction is particularly important because the existing systems were developed for different clinical purposes and should not be considered interchangeable.

For treatment decision-making, the SITE Score currently has the most extensive independent evaluation among the disease-specific tools identified in this review [15,16,17,18]. Its performance across external cohorts has nevertheless varied from moderate to good, and its most recent independent evaluation demonstrated only moderate discrimination [18]. BSDS provides a contrasting example: despite favorable performance during development [14], subsequent studies produced markedly heterogeneous results, including failure to discriminate operative from non-operative management in an independent population [30,32,33]. These findings illustrate the importance of external validation before a treatment-decision score is adopted beyond the setting in which it was developed.

A key limitation is the nature of the outcome used to evaluate treatment-decision scores. Most studies have assessed whether a score discriminates between patients who were actually managed surgically and those treated conservatively, or whether its recommendation agrees with expert clinical judgment [15,16,17,18]. Neither endpoint necessarily represents an objective determination that surgery was required. Operative management may be influenced by neurological status, disease severity, patient comorbidity, local practice patterns, surgeon preference, and other factors not fully captured by a scoring system. Consequently, prediction of observed surgical management should be distinguished from prediction of an independently adjudicated need for surgery. This limitation is particularly relevant when interpreting apparently high discrimination for treatment-decision tools.

For severity and mechanical instability, the evidence addresses a different clinical construct. The Pola classification has demonstrated independent inter-observer reliability [21] and has also been evaluated against operative management [17], whereas SISS and particularly mSISS have shown good-to-excellent reproducibility for the assessment of infection-related instability [22,23,34]. However, SISS performed poorly when subsequently evaluated for discrimination of operative versus non-operative management [16]. This illustrates a central methodological point: a tool may be reliable for the construct it was designed to assess while having limited ability to predict a broader clinical decision. MRI-SSI similarly provides a structured imaging-based assessment of disease severity and showed moderate discrimination for subsequent operative management in its development cohort [24], but independent external validation remains lacking.

Diagnostic and longitudinal assessment tools should likewise be interpreted according to their intended purpose. The SPONDY-Score was developed to distinguish spondylodiscitis from degenerative spinal disease and has undergone internal testing, but not independent external validation [9]. SponDT integrates inflammatory activity, pain, and MRI findings and is primarily intended for disease assessment and longitudinal treatment monitoring [26,35], while SSC combines clinical, laboratory, and radiological parameters within a severity and treatment-guidance framework [25,36]. The absence of external outcome validation for these instruments should not be interpreted as evidence that they lack clinical usefulness; rather, it limits the conclusions that can be drawn regarding their generalizability and predictive performance.

The evidence for mortality prediction remains comparatively limited. MSI-20 represents a dedicated mortality risk-stratification tool, but it was developed in a broader population of patients with spinal infection rather than exclusively in pyogenic spondylodiscitis and has not yet undergone independent external validation [10,28,37,38,39,40]. Other studies have identified individual predictors of adverse outcome or mortality, including comorbidity burden, neurological impairment, delayed diagnosis, inflammatory response, and imaging evidence of neural compression [27,29,41]. Thus, the literature on prognostic factors remains considerably more developed than the literature on externally validated multivariable prognostic scores.

Taken together, these findings suggest that no single currently available tool can be considered comprehensively validated across disease severity assessment, prognosis, and treatment decision-making. This should not be interpreted as evidence that existing scores are clinically uninformative. Rather, each tool should be used, where appropriate, within the clinical construct for which it was developed and with recognition of the specific type of validation supporting it. Future studies should prioritize independent multicenter validation, clearly predefined outcomes, assessment of both discrimination and calibration, and evaluation of whether score-based decisions provide incremental value beyond routine multidisciplinary clinical assessment. For treatment-decision models in particular, future validation should also address the methodological challenge of defining an appropriate reference standard for surgical necessity rather than relying exclusively on treatment actually delivered.

## 5. Conclusions

Several scoring and classification systems have been developed for pyogenic spondylodiscitis, addressing distinct clinical purposes including diagnosis, disease-severity assessment, mechanical instability, treatment selection, longitudinal monitoring, and mortality prediction. However, the level and type of validation supporting these tools vary substantially. Reliability, internal performance, and external predictive validation should not be considered interchangeable, and favorable results in development cohorts have not always been reproduced in independent populations.

Among treatment-decision tools, the SITE Score currently has the most extensive independent evaluation, although its performance remains variable across cohorts and available studies predominantly predict observed surgical management rather than an independently established need for surgery. Other systems, including BSDS, Pola, SISS/mSISS, MRI-SSI, SponDT, SSC, SPONDY-Score, and MSI-20, provide potentially useful information within their respective clinical domains but have important limitations in external validation or outcome-based assessment.

At present, no single scoring system can be recommended as a comprehensive or definitive decision tool for pyogenic spondylodiscitis. Existing scores are best regarded as adjuncts to clinical and multidisciplinary assessment, used according to the specific construct for which they were developed. Future research should prioritize prospective, multicenter external validation using clearly defined and clinically meaningful outcomes before widespread implementation of these tools can be supported.

## Figures and Tables

**Table 1:** Development and validation evidence for scoring and classification systems used in pyogenic spondylodiscitis.

Tool	Primary Purpose	Development/ Internal Evidence	Independent Evidence	Type of Validation	Main Conclusion
BSDS	Treatment decision	AUC 0.83; additional cohort AUC 0.71 [14]	AUC 0.73 [17]; 0.469 [30]	Predictive	Markedly variable external performance
SITE	Treatment decision	Proof-of-concept; high agreement/reliability [15]	AUC 0.743 [16]; 0.86 [17]; 0.70 [18]	Predictive	Most extensively externally evaluated treatment-decision tool; performance remains variable
Pola	Severity/ treatment classification	Development and clinical evaluation in 250 patients [20]	Inter-observer reliability [21]; AUC 0.81 [17]	Reliability + predictive	Reproducible classification; limited external predictive evidence
SISS	Mechanical instability	Excellent inter-observer reliability; ICC 0.960 [22]	ICC 0.68; AUC 0.557 for operative management [16]	Reliability + predictive	Reproducible for instability; poor prediction of operative management
mSISS	Mechanical instability	Expert-consensus modification; multicenter reliability assessment [23]	None identified	Reliability	High reproducibility; outcome-based predictive validity not established
MRI-SSI	Imaging-based severity	AUC 0.72 for subsequent surgery [24]	None identified	Development-cohort predictive assessment	Moderate initial discrimination; external validation lacking
SPONDY-Score	Diagnosis	Internal train/test evaluation [9]	None identified	Internal diagnostic testing	Promising diagnostic performance; independent validation lacking
SponDT	Disease assessment/monitoring	Development/descriptive evidence [26]	None identified	Development/clinical application	External outcome validation lacking
SSC	Severity/ treatment guidance	Development/clinical application [25]	None identified	Development evidence	Limited validation
MSI-20	Mortality prediction	Retrospective development cohort [28]	None identified	Development-cohort prognostic assessment	Promising mortality stratification; external validation lacking

**Table 2:** Main purposes and variables incorporated into scoring systems and classifications evaluated in spondylodiscitis.

Scoring System/Classification	Primary Purpose	Main Variables or Domains
**Brighton Spondylodiscitis Score (BSDS)** [14]	Estimation of the likelihood of surgical intervention	Distant-site infection; medical comorbidities; immunocompromised status; MRI findings, including non-specific fluid collection, vertebral collapse, and abscess formation; anatomical location; neurological impairment.
**Spinal Infection Treatment Evaluation (SITE) Score** [15]	Treatment decision support in de novo spinal infection	Neurological status; anatomical location; radiological findings, including osseous destruction, deformity or translation, posterolateral involvement, and neural-element compression; pain severity and its effect on ambulation; host comorbidities.
**Spinal Instability Neoplastic Score (SINS)** [19]	Assessment of mechanical instability in neoplastic spinal disease; evaluated as a comparator in spinal infection	Anatomical location; mechanical pain; bone lesion type; spinal alignment; vertebral body collapse; posterolateral involvement of spinal elements.
**Pola classification** [20]	Clinical–radiological classification and treatment stratification in pyogenic spondylodiscitis	Disc and vertebral involvement; bone destruction; biomechanical instability and segmental kyphosis; paravertebral soft-tissue involvement or intramuscular abscesses; epidural abscess; neurological impairment.
**Spinal Instability Spondylodiscitis Score (SISS)** [22]	Assessment of infection-related mechanical instability	Extent of bone involvement; spinal alignment; anatomical location; mechanical pain.
**Modified Spinal Instability Spondylodiscitis Score (mSISS)** [23]	Simplified assessment of infection-related mechanical instability	Anatomical location; extent of bone lesion at the affected spinal segment; spinal alignment; mechanical pain.
**MRI Spondylodiscitis Severity Index (MRI-SSI)** [24]	MRI-based assessment of disease severity and exploration of its association with subsequent surgery	Vertebral destruction and height loss; vertebral signal abnormalities; endplate erosion; disc abnormalities; posterior vertebral involvement; paravertebral and epidural extension or abscess formation.
**Spondylodiscitis Severity Code (SSC)** [25,26]	Severity classification and treatment stratification	SponDT-based disease activity assessment, incorporating CRP, pain, and MRI findings; neurological status; radiological evidence of vertebral destruction.
**SPONDY-Score** [9]	Differentiation of infectious from degenerative vertebral endplate abnormalities on MRI	Posterior-element bone marrow edema; vertebral body destruction; paravertebral edema; disc T2 hypointensity, assigned a negative weight; non-anatomical disc T2 hyperintensity.
**Spondylodiscitis Diagnosis and Treatment (SponDT) score** [26]	Assessment of disease activity and longitudinal monitoring during treatment	Serum CRP; pain intensity measured using an NRS; MRI findings categorized as no active changes/residual changes, discitis, spondylodiscitis, or spondylodiscitis with abscess.
**Mortality in Spinal Infection (MSI-20) score** [28]	Mortality risk stratification in spinal infection	BMI; ASA physical status; sepsis; age-adjusted Charlson Comorbidity Index; renal insufficiency; hepatopathy; neurological impairment assessed through muscle strength; CRP at diagnosis.

Abbreviations: ASA, American Society of Anesthesiologists; BMI, body mass index; CRP, C-reactive protein; MRI, magnetic resonance imaging; NRS, numerical rating scale.

**Table 3:** Main advantages and limitations of scoring systems and classifications evaluated in spondylodiscitis.

Scoring System/Classification	Main Advantages	Main Limitations and Shortcomings
**Brighton Spondylodiscitis Score (BSDS)** [14,17,30]	Developed specifically for spondylodiscitis; combines clinical, neurological, host-related, and MRI parameters; provides a numerical estimate of the likelihood of surgical intervention.	Derived from a small retrospective cohort; markedly variable performance across external cohorts; may be influenced by population characteristics and local treatment practices; prediction of actual surgical management does not establish the necessity or benefit of surgery.
**Spinal Infection Treatment Evaluation (SITE) Score** [15,16,17,18,19]	Integrates neurological, radiological, functional, and host-related factors; provides a structured framework for conservative versus surgical management; demonstrated good interobserver agreement and has been evaluated in several independent cohorts.	Discriminatory performance and operating characteristics vary across cohorts; some radiological categories require interpretive judgment; validation primarily concerns actual treatment or expert recommendations rather than independently established surgical necessity; patient preferences are not explicitly incorporated.
**Spinal Instability Neoplastic Score (SINS)** [19]	Widely used in neoplastic spinal disease; provides a structured assessment of mechanical instability using familiar clinical and imaging variables; offers a comparator for infection-specific instability tools.	Developed for neoplastic rather than infectious disease; tumor-specific bone-lesion categories limit applicability to spondylodiscitis; does not incorporate inflammatory activity, abscess burden, neurological compromise, or systemic illness; cannot independently guide treatment selection in spinal infection.
**Pola classification** [17,20,21]	Developed specifically for pyogenic spondylodiscitis; integrates structural destruction, mechanical instability, paravertebral and epidural involvement, and neurological status; provides treatment-oriented categories; independent reproducibility has been assessed.	Multiple subtypes increase classification complexity; original development and clinical evaluation used the same cohort; independent predictive evidence remains limited; host comorbidities and systemic inflammatory activity are not explicit classification domains; further prospective outcome validation is required.
**Spinal Instability Spondylodiscitis Score (SISS)** [16,22]	Specifically designed to assess infection-related mechanical instability; uses a focused four-domain framework; demonstrated excellent interobserver reliability in the original study.	Primarily assesses mechanical instability rather than overall disease severity; does not directly incorporate neurological status, systemic illness, or inflammatory activity; showed poor discrimination for operative management in an independent cohort; does not encompass all indications for surgery.
**Modified Spinal Instability Spondylodiscitis Score (mSISS)** [23]	Simplifies SISS definitions and assesses bone involvement at the affected spinal segment; provides a structured instability classification; demonstrated high inter- and intraobserver reliability in a multicenter assessment.	Reliability assessment involved a limited set of representative cases evaluated by experienced surgeons; predictive validity for clinical outcomes or operative management remains unestablished; retains a narrow focus on mechanical instability.
**MRI Spondylodiscitis Severity Index (MRI-SSI)** [24]	Provides a detailed, quantitative MRI-based assessment of disease extent; incorporates vertebral, disc, paravertebral, and epidural involvement; demonstrated an initial association with subsequent surgical treatment.	Developed in a small retrospective single-center cohort; moderate initial discrimination and limited sensitivity at the proposed severe-disease threshold; requires detailed contrast-enhanced MRI assessment; does not incorporate clinical status or host comorbidities; independent external validation remains lacking.
**Spondylodiscitis Severity Code (SSC)** [25,26]	Combines clinical and laboratory disease activity with neurological status and structural destruction; links severity categories to proposed treatment strategies.	Conservatively treated patients were excluded from the original classification analysis, limiting assessment across the full treatment spectrum; independent outcome validation remains limited; evidence predominantly derives from retrospective development and clinical application; benefit from implementing the proposed treatment pathways has not been established.
**SPONDY-Score** [9]	Uses defined MRI features to differentiate infectious from degenerative vertebral endplate abnormalities; demonstrated promising diagnostic performance and imaging-feature reproducibility during initial evaluation.	Primarily diagnostic rather than prognostic or treatment-oriented; testing used a random split of the original dataset rather than an independent population; reported diagnostic performance also incorporated paravertebral collections outside the five-item score; independent external validation remains required.
**Spondylodiscitis Diagnosis and Treatment (SponDT) score** [26]	Simple composite score combining pain, inflammatory activity, and MRI findings; supports longitudinal assessment of disease activity during treatment.	Covers a limited number of domains; pain assessment is subjective; repeated MRI limits practicality, and imaging abnormalities may persist despite clinical improvement; does not directly assess neurological deficit, comorbidity burden, or mechanical instability; independent validation against predefined clinical outcomes remains lacking.
**Mortality in Spinal Infection (MSI-20) score** [28,31]	Specifically developed for mortality risk stratification; uses routinely collected clinical and laboratory parameters; incorporates systemic illness, comorbidity burden, and neurological function; recently evaluated in a large multicenter external cohort.	Retrospective evidence and moderate discrimination limit individual risk prediction; designed for mortality stratification rather than anatomical severity assessment or treatment selection; prospective clinical utility requires further evaluation; should not independently determine surgical eligibility or the relative benefit of operative versus conservative treatment.

Abbreviations: MRI, magnetic resonance imaging.

## Data Availability

No new data were created or analyzed in this study. Data sharing is not applicable to this article.

## Supplementary Materials

The following supporting information can be downloaded at https://www.mdpi.com/article/10.3390/diagnostics16213511/s1. Supplementary File S1: Declaration of AI-Assisted Technology Use.

## Abbreviations

The following abbreviations are used in this manuscript:

AUC	Area under the receiver operating characteristic curve
BSDS	Brighton Spondylodiscitis Score
CI	Confidence interval
CRP	C-reactive protein
ICC	Intraclass correlation coefficient
MRI-SSI	MRI Spondylodiscitis Severity Index
mSISS	Modified Spinal Instability Spondylodiscitis Score
MSI-20	Mortality in Spinal Infection score
NRS	Numerical rating scale
SINS	Spinal Instability Neoplastic Score
SISS	Spinal Instability Spondylodiscitis Score
SITE	Spinal Infection Treatment Evaluation Score
SponDT	Spondylodiscitis Diagnosis and Treatment score
SSC	Spondylodiscitis Severity Code
