# Supplementary material for: Scoring Systems in Pyogenic Spondylodiscitis—A Narrative Review
Source: Diagnostics (Basel). 2026 Oct 27;16(21):3511. doi: 10.3390/diagnostics16213511 (PMC13761946; doi:10.3390/diagnostics16213511)
Supplement: Supplementary file 1 [file diagnostics-16-03511-s001.zip › diagnostics-4572172-supplementary.pdf]

Supplementary Material [S1] – Declaration of AI-Assisted Technology Use

- **Tool used:** [e.g., ChatGPT, OpenAI, Claude], accessed [august/2026].
- **Purpose:** Language assistance only. Used to find suitable English wording for individual terms and short expressions.
- **Input:** Single words or short phrases written by the authors, with a request for alternative English wording. No full sentences, paragraphs, manuscript sections or study data were entered.
- **Output:** Alternative wording suggestions for these terms and phrases.
- **Human review:** The authors checked each suggestion for accuracy and fit with the scientific context. They accepted, modified, or rejected each one. The final text was written and approved by all authors.
- **Not used for:** Study design, data collection, statistical analysis, interpretation of results, tables, figures, references, or drafting of manuscript text.
- **Responsibility:** The authors take full responsibility for the content and integrity of the manuscript.
